# Acetylated DNMT1 Downregulation and Related Regulatory Factors Influence Metastatic Melanoma Patients Survival

**DOI:** 10.3390/cancers13184691

**Published:** 2021-09-18

**Authors:** Xiaoqing Zhang, Matias A. Bustos, Yoshiaki Shoji, Romela Irene Ramos, Yuuki Iida, Rebecca Gentry, Teh-Ling Takeshima, Dave S. B. Hoon

**Affiliations:** Department of Translational Molecular Medicine, Saint John’s Cancer Institute (SJCI), Providence Saint John’s Health Center (SJHC), Santa Monica, CA 90404, USA; Xiaoqing.Zhang@providence.org (X.Z.); BustosM@jwci.org (M.A.B.); ShojiY@jwci.org (Y.S.); Romela.Ramos@providence.org (R.I.R.); yiida-tky@umin.ac.jp (Y.I.); Rebecca.Gentry@providence.org (R.G.); teh-ling.takeshima@providence.org (T.-L.T.)

**Keywords:** acetylated-DNMT1, cutaneous metastatic melanoma, DNMT1, panobinostat, TIP60, USP7

## Abstract

**Simple Summary:**

DNA methyltransferase-1 (DNMT1) is a key epigenetic regulatory protein of gene expression in cutaneous melanoma. DNMT1 is acetylated by TIP60 promoting its degradation. This study demonstrated that DNMT1 and ac-DNMT1 protein levels were inversely correlated in stage III (*n* = 17) and stage IV (*n* = 164) metastatic melanoma tumors, and both influenced melanoma progression. Reduced TIP60 and USP7 protein expression levels were correlated with decreased ac-DNMT1 levels. Of clinical translational relevance, patients with high ac-DNMT1 protein levels, or high-acDNMT1 with concurrent low DNMT1, high TIP60, or high USP7 protein levels showed significantly better prognosis for 4-year melanoma-specific survival. These results suggested that ac-DNMT1 is a significant post-translational modification influencing advanced melanoma patient disease outcomes.

**Abstract:**

The role of post-translational modifications (PTM) of the key epigenetic factor DNMT1 protein has not been well explored in cutaneous metastatic melanoma progression. The acetylated DNMT1 (ac-DNMT1) protein level was assessed using an anti-acetylated lysine antibody in a clinically annotated melanoma patient tumor specimen cohort. In this study, we showed that surgically resected tumors have significantly higher DNMT1 protein expression in metastatic melanoma (stage III metastasis *n* = 17, *p* = 0.0009; stage IV metastasis *n* = 164, *p* = 0.003) compared to normal organ tissues (*n* = 19). Additionally, reduced ac-DNMT1 protein levels were associated with melanoma progression. There was a significant inverse correlation between ac-DNMT1 and DNMT1 protein levels in stage IV metastatic melanoma (*r* = −0.18, *p* = 0.02, *n* = 164). Additionally, ac-DNMT1 protein levels were also significantly positively correlated with TIP60 (*r* = 0.6, *p* < 0.0001) and USP7 (*r* = 0.74, *p* < 0.0001) protein levels in stage IV metastatic melanoma (*n* = 164). Protein analysis in metastatic melanoma tumor tissues showed that with high ac-DNMT1 (*p* = 0.006, *n* = 59), or concurrent high ac-DNMT1 with low DNMT1 (*p* = 0.05, *n* = 27), or high TIP60 (*p* = 0.007, *n* = 41), or high USP7 (*p* = 0.01, *n* = 48) consistently showed better 4-year melanoma-specific survival (MSS). Multivariate Cox proportional hazard analysis showed that ac-DNMT1 level is a significant independent factor associated with MSS (HR, 0.994; 95% confidential interval (CI), 0.990–0.998; *p* = 0.002). These results demonstrated that low ac-DNMT1 levels may represent an important regulatory factor in controlling metastatic melanoma progression and a promising factor for stratifying aggressive stage IV metastasis.

## 1. Introduction

Cutaneous malignant melanoma is a deadly skin cancer, accounting for the highest skin cancer deaths worldwide with an increasing rate of incidence [1]. Cutaneous melanoma has a high propensity to metastasize to multiple distant organs if not diagnosed and treated early [1], and predominantly affects the Caucasian population in North America, Australia, and Europe [2,3]. Cutaneous melanoma causes approximately 60,000 deaths worldwide each year [4]. Patients with AJCC stage IV melanoma have a very poor prognosis, whereby the general 5-year overall survival rate of patients is <25% [5]. Currently, the National Comprehensive Cancer Network (NCCN) recommends checkpoint inhibitor immunotherapies (nivolumab, ipilimumab, pembrolizumab, and others) for the treatment of stage IV melanoma patients [6,7]. To date, the overall survival of stage IV metastatic melanoma patients has significantly improved from modern approved checkpoint inhibitor immunotherapies and targeted therapies [8,9,10]. However, durable survival is still poor with variable responses amongst patients. Predictable factors for stage IV metastasis are very limited. Melanoma patients with stage IV are very heterogeneous population in disease outcomes, whereby some patients have longer survival than others. To further improve the outcomes of these patients, there is an urgent need to identify new prognostic biomarkers that will help to better stratify patients and decide the best systemic adjuvant or neoadjuvant immunotherapy for metastatic melanoma.

Abnormal genomic methylation status of genes is strongly correlated with cutaneous melanoma development and progression through regulating the expression of various tumor suppressor genes and oncogenes [11,12,13]. Mammalian DNA methylation patterns are catalyzed by key DNA methyltransferases (DNMT), which include DNMT1, DNMT3a, and DNMT3b in human cells [14]. DNMT3a and DNMT3b establish de novo DNA-methylation levels during embryonic development and adult tissue differentiation [15,16]; whereas, DNMT1 propagates DNA-methylation genomic patterns to daughter cells during replication at the S phase [17,18]. The DNMT3 family plays an important role in melanoma tumor progression [19,20,21]; however, the role of DNMT1 is less understood. To date, limited studies have investigated the expression of DNMT1 in melanoma patients and disease outcomes [22]. Assessment of nevus, primary melanoma, and melanoma metastasis show that DNMT1 expression is enhanced with melanoma progression and positively correlates with the Ki-67 levels [22]. Several studies show that suppressed DNMT1 expression caused cell cycle delay and cancer-germline gene activation [22,23,24,25]. However, it is unclear how DNMT1 protein regulators and post-translational modifications determine DNMT1 expression to influence metastatic melanoma outcomes.

The regulation of DNMT1 is controlled by specific post-translational modifications (PTM), such as acetylation and ubiquitination [26,27,28,29]. Ubiquitination of DNMT1 is important for its degradation to reduce DNMT1 levels once the cell cycle is completed [29]. Investigating the aberrant mechanisms that control DNMT1 activity during melanoma progression may improve our understanding of gene methylation during tumor progression. Acetylation modification on a protein can change its solubility, surface properties, and hydrophobicity, which will influence enzymatic activity, stability, protein–DNA interactions, and protein–protein interactions [30,31]. The lysine-acetyltransferase Tat-interactive protein 60 (TIP60, also known as lysine acetyltransferase 5) is downregulated in multiple cancers [32,33]. TIP60 controls the acetylation on specific lysine residues of DNMT1 regulating its stability and activity [26,34,35]. In addition, Cheng et al. found that DNMT1 acetylation by TIP60 on four lysine residues within the KG linker impairs DNMT1 and ubiquitin-specific protease 7 (USP7) interaction and triggers the proteasomal degradation of DNMT1 [34].

In this study, we assessed DNMT1 and acetylated-DNMT1 (ac-DNMT1) protein levels in AJCC stage III and IV melanoma patients as related to disease progression and in comparison to normal organ control tissues. Tissues from stage III and IV melanoma patients showed reduced protein levels of ac-DNMT1 and consequently enhanced protein expression of DNMT1 compared to primary melanoma tumors and normal organ control tissues. A significant correlation was observed between ac-DNMT1 and DNMT1/TIP60/USP7 protein levels. Additionally, decreased ac-DNMT1 protein levels were associated with melanoma progression. Patients with high ac-DNMT1, or patients with high ac-DNMT1 and concurrent low DNMT1, high TIP60, or high USP7 protein levels consistently had better survival status. Multivariate analysis indicated that ac-DNMT1 protein level was an independent factor for melanoma-specific survival (MSS). This study demonstrated that TIP60 and USP7 downregulation decreases ac-DNMT1 protein levels, which increased the amount of DNMT1 and significantly influenced stage IV melanoma disease outcomes.

## 2. Materials and Methods

### 2.1. Melanoma Tissue Microarray (TMA)

The TMA (336 samples from 170 patients) was developed at Saint John’s Cancer Institute (SJCI) using clinicopathological annotated AJCC 8th Ed stage III and IV formalin-fixed paraffin-embedded (FFPE) melanoma tissues [36,37]. The TMA was predominately enriched with stage IV tissues (19 stage III and 287 stage IV). Stage III tissues were autologous paired to the stage IV tissues. These tissue samples were accessioned at Saint John’s Health Center (SJHC) pathology department with annotated clinical outcomes and follow-up data using the SJCI melanoma database. In this study, TMAs were assessed for protein levels of DNMT1, ac-DNMT1, Ki-67, TIP60, and USP7 by IHC and H-score [36]. The cores in the TMA were spread out in duplicates randomly to avoid spatial bias from IHC staining, whereby the H-score average was calculated in duplicates. Due to loss of cores from sectioning, there were a total of 200 evaluable samples from the TMA which included: 19 normal organs (2 adrenal, 2 bowel, 1 brain, 2 gallbladder, 2 kidney, 3 liver, 1 lung, 2 ovary, 2 pancreas, 2 spleen), 17 stage III, and 164 stage IV metastatic tissues for analysis. For patient stratification into low and high groups, the mean H-score was selected as the cut-off value. The clinicopathological characteristics of the stage IV melanoma patients that were divided based on the mean H-score for ac-DNMT1, DNMT1, TIP60, or USP7 are listed in Table S1.

### 2.2. Immunohistochemistry (IHC)

IHC staining was performed as previously described [38,39], using anti-human DNMT1 rabbit polyclonal antibody (Ab, 1:100 dilution, Cat# NB100-264, Novus Biologicals, Centennial, CO, USA), anti-human ac-DNMT1 rabbit polyclonal Ab (1:100 dilution, Cat# A5595, ABclonal, Woburn, MA, USA), anti-human Ki-67 mouse monoclonal Ab (1:100 dilution, Cat# M7240, DAKO, Santa Clara, CA, USA), anti-human TIP60 rabbit polyclonal Ab (1:100 dilution, Cat# 10827-1-AP, Proteintech, Rosemont, IL, USA), and anti-human USP7 rabbit monoclonal Ab (1:100 dilution, Cat# A700-072, Bethyl, Montgomery, TX, USA) for the TMA slide. Additionally, for initial testing, we performed IHC for ac-DNMT1 on 8 primary, 15 stage III, and 15 stage IV metastatic melanoma FFPE tissue slides of clinically annotated specimens from SJHC pathology. Images were taken with a BX43 upright microscope (Olympus, Tokyo, Japan) at 20× magnification and analyzed using inForm 2.4 software (Perkin Elmer, Waltham, MA, USA). H-scores were calculated following the inForm software instructions available at https://www.perkinelmer.com/Content/LST_SoftwareDownloads/inFormUserManual_2_3_0_rev1.pdf (accessed on 5 May 2021).

### 2.3. Melanoma Cell Lines

Established metastatic melanoma cell lines (MH-0331 and WP-0614) from SJCI were attained from melanoma patients who received elective surgery at SJHC [40]. The cell lines were cultured in RPMI-1640 and supplemented with 10 mM HEPES, 10% heat-inactivated fetal bovine serum (FBS), and 1% penicillin–streptomycin (complete medium) as previously described. All human cell lines have been authenticated using short tandem repeat (STR) profiling within the last three years. All experiments were performed with mycoplasma-free melanoma cell lines.

### 2.4. Western Blot Analysis

Two melanoma cell lines (MH-0331 and WP-0614) were treated for 24 h with HDAC inhibitor panobinostat (Cat# HY-10224, MedChemExpress, Monmouth Junction, NJ, USA) at different concentrations (10, 50, and 100 nM). Protein was then extracted and evaluated by Western blot for DNMT1, ac-DNMT1, and β-actin as previously described [38,41], except for the Abs utilized, which were the polyclonal rabbit anti-human DNMT1 Ab (1:100 dilution, Cat# NB100-264, Novus Biologicals), the rabbit anti-human ac-DNMT1 Ab (1:500 dilution, Cat# A5595, ABclonal), and the mouse monoclonal anti-human β-actin Ab (1:10000 dilution, Cat# A5441, Sigma-Aldrich). All Western blot images were analyzed with ImageJ software (http://imagej.nih.gov/ij/ (accessed on 3 August 2021). All the uncropped Western blot images were included in Figure S1.

### 2.5. Biostatistical Analysis

The statistical analyses were performed using GraphPad Prism 7 software (GraphPad software Inc., La Jolla, CA, USA) with two-tailed tests. The distribution and variation within each group of data was assessed before selecting the correct statistical analysis. Multiple groups were analyzed by one-way or two-way ANOVA followed by post-hoc tests. The correlation was determined by the Spearman’s or Pearson’s correlation test. The 4-year melanoma-specific survival (MSS) was calculated from the time of the patient’s initial diagnosis as stage IV until death or last contact. MSS was analyzed using the Kaplan–Meier method and log-rank test. For 15 melanoma patients who had two different surgery dates for the melanoma metastatic tissues, the clinical information pertaining to the first surgery/distant metastasis was used for survival analysis. Univariate and multivariate analyses were performed using SPSS software (IBM, Armonk, NY, USA). A *p* < 0.05 is considered significant if not stated otherwise. All the figures were unified using Adobe Illustrator CC (Adobe Inc., Los Angeles, CA, USA).

## 3. Results

### 3.1. DNMT1 Expression Is Increased in Metastatic Melanoma

**Figure 1:**
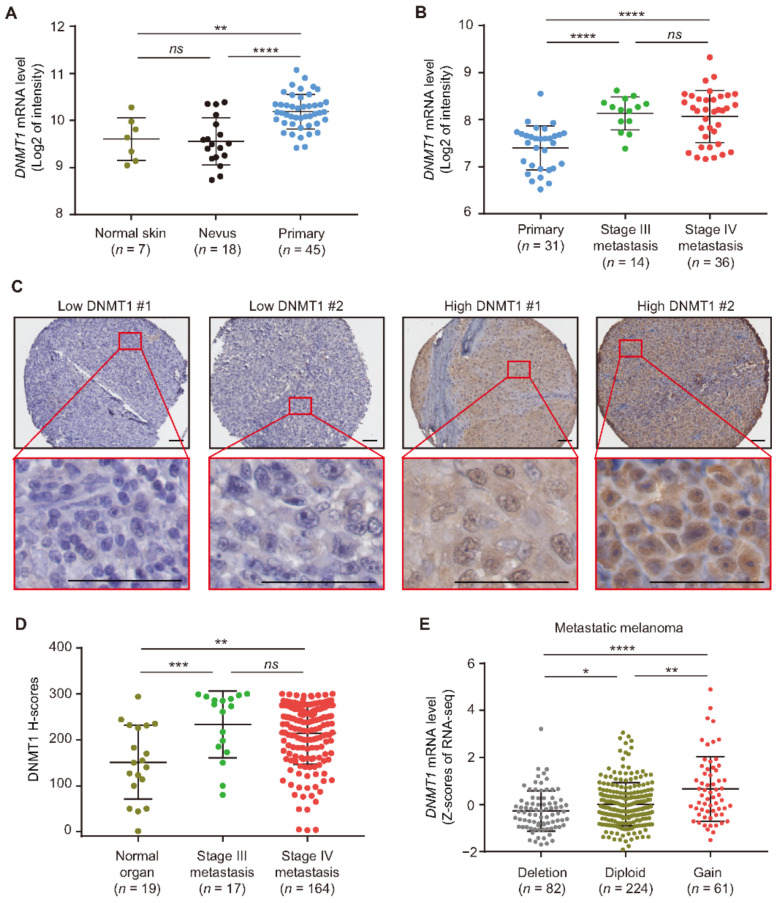
DNMT1 expression is enhanced in metastatic melanoma. (**A**) Comparison of *DNMT1* mRNA expression in normal skin, nevus, and primary melanoma samples using GSE3189 microarray dataset. (**B**) Comparison of *DNMT1* mRNA expression in primary melanoma, stage III, and stage IV metastatic melanoma tissues using the GSE8401 microarray dataset. (**C**,**D**) Representative IHC images and H-score quantification of DNMT1 in normal organ tissues, stage III, and stage IV metastatic melanoma tissues from the TMA cohort. Scale bars = 20 μm. (**E**) Distribution of *DNMT1* mRNA levels in metastatic melanoma patients with hemizygous deletion, diploid, and gain using the TCGA metastatic melanoma dataset (*n* = 367). Data represent the mean ± SD. *ns*: not significant, * *p* < 0.05, ** *p* < 0.01, *** *p* < 0.001, and **** *p* < 0.0001.

To investigate the *DNMT1* mRNA expression in early and advanced stage cutaneous melanoma, we interrogated two FFPE tissue microarray datasets (GSE3189 and GSE8401). The first microarray dataset GSE3189 contained normal skin, benign nevus, and primary melanoma tumor tissues. *DNMT1* mRNA levels were significantly increased in primary melanoma tumors compared to nevus (*p* < 0.0001) or normal skin tissues (*p* = 0.003; Figure 1A). The second tissue microarray GSE8401 contained primary and metastatic melanoma tissues. Metastatic melanoma showed an increase in *DNMT1* mRNA expression compared to primary melanoma (*p* < 0.0001; Figure 1B). To further validate the observation of the increased DNMT1 expression in melanoma, we performed IHC for DNMT1 on our SJCI-established TMA sections that contained melanoma and normal organ FFPE tissues. Two patients with low or high DNMT1 protein levels are shown in Figure 1C. There were significantly higher DNMT1 protein levels in stage III (*p* = 0.0009) and stage IV (*p* = 0.003) melanoma patients compared to normal organ tissues (Figure 1D). These findings indicate that *DNMT1* mRNA is enhanced during melanoma progression. Furthermore, significantly higher DNMT1 protein expression was observed in metastatic melanoma.

To determine factors that may be associated with enhanced DNMT1 expression, we investigated the copy number variation (CNV) dataset of metastatic melanoma patients from the TCGA SKCM project. There was a significant correlation between *DNMT1* CNV and mRNA expression levels (*r* = 0.21, *p* < 0.0001, Figure 1E). However, only 16.6% (*n* = 61) of metastatic melanoma samples had a gain in CNV for *DNMT1* (Figure 1E). These results suggest that CNV partially explains the overall increase of *DNMT1* mRNA levels in metastatic melanoma. Thus, there may be other regulatory mechanisms enhancing DNMT1 expression or promoting its protein stability in metastatic melanoma, such as specific PTMs.

### 3.2. Increased Ac-DNMT1 Leads to a Reduction in DNMT1 Protein Levels

**Figure 2:**
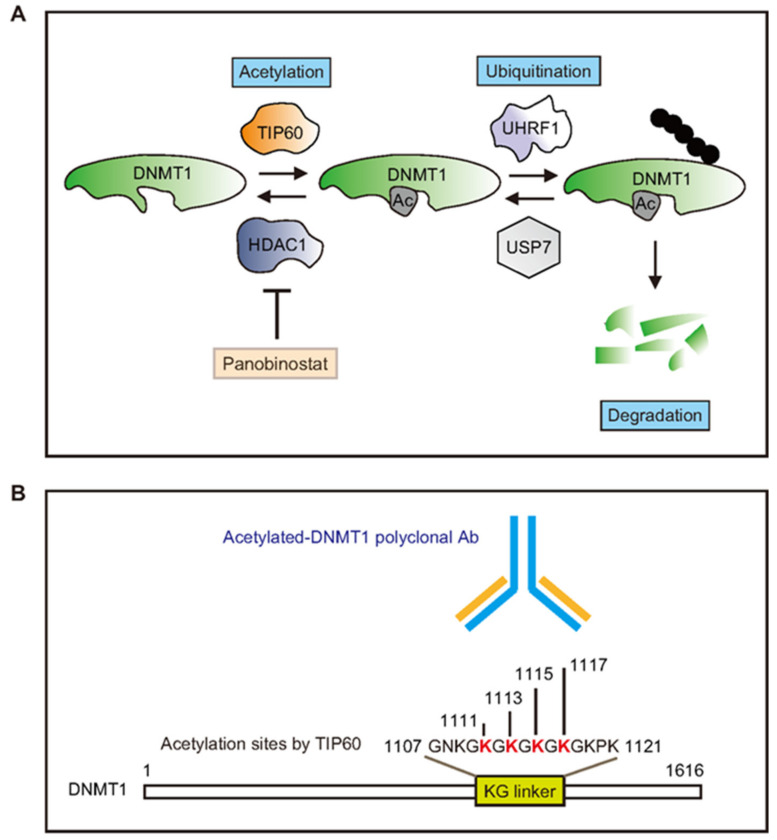

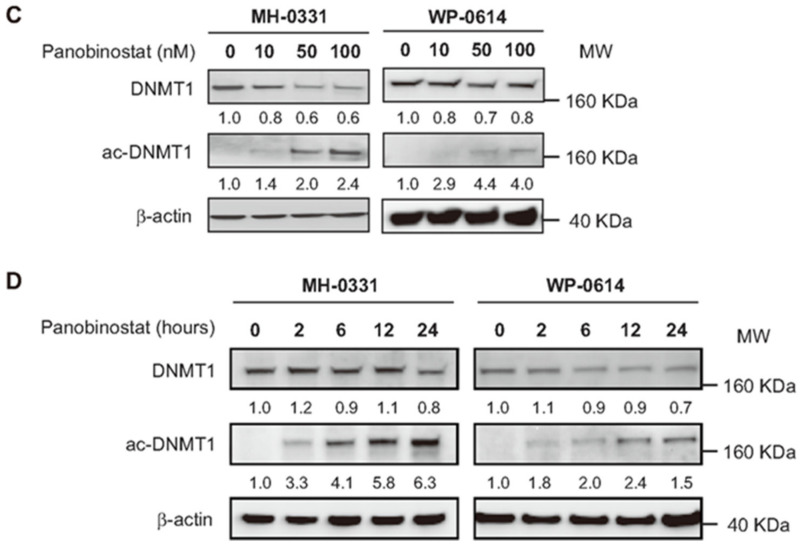
Molecular mechanisms controlling DNMT1 PTMs and protein stability. (**A**) Schematic diagram showing the acetylation process that modulates the stability of DNMT1 protein. (**B**) The ac-DNMT1 Ab recognizes the four acetylated lysine residues located in the KG linker of the DNMT1 protein domain. (**C**,**D**) Western blot of DNMT1 and ac-DNMT1 in metastatic melanoma cells treated with different concentrations (10, 50, and 100 nM) of panobinostat for 24 h (**C**) or treated with 100 nM panobinostat (**D**) for the indicated times (2, 6, 12, and 24 h). β-actin was used as the loading control.

DNMT1 acetylation, which can induce ubiquitin-dependent proteasomal degradation (Figure 2A), is an important PTM that controls DNMT1 protein stability [26,34]. TIP60 regulates DNMT1 acetylation, while histone deacetylase 1 (HDAC1) controls DNMT1 deacetylation. Ac-DNMT1 protein levels were evaluated by the ac-DNMT1 Ab which specifically targets the four lysine residues (K1111/K1113/K1115/K1117) within the KG linker (1107GNKGKGKGKGKGKPK1121, Figure 2B). To further determine the role of acetylation on DNMT1 protein stability, we evaluated the effect of panobinostat treatment in metastatic melanoma cell lines. Panobinostat is a pan-inhibitor for multiple HDAC proteins [42,43,44], which can block HDAC1 and the deacetylation of various proteins including DNMT1 (Figure 2A). Metastatic melanoma cell lines were treated with different concentrations of panobinostat (0, 10, 50, and 100 nM for 24 h; Figure 2C). We determined the optimal concentration and then treated metastatic melanoma cell lines with 100 nM panobinostat at different time points (0, 2, 6, 12, 24 h; Figure 2D). Panobinostat treatment enhanced ac-DNMT1 protein levels with a concomitant reduction in DNMT1 protein levels in both melanoma cell lines (Figure 2C,D). These results suggested that acetylation plays a major role in controlling DNMT1 stability and that there may be an inverse correlation between ac-DNMT1 and total DNMT1 protein levels in melanoma patients.

**Figure 3:**
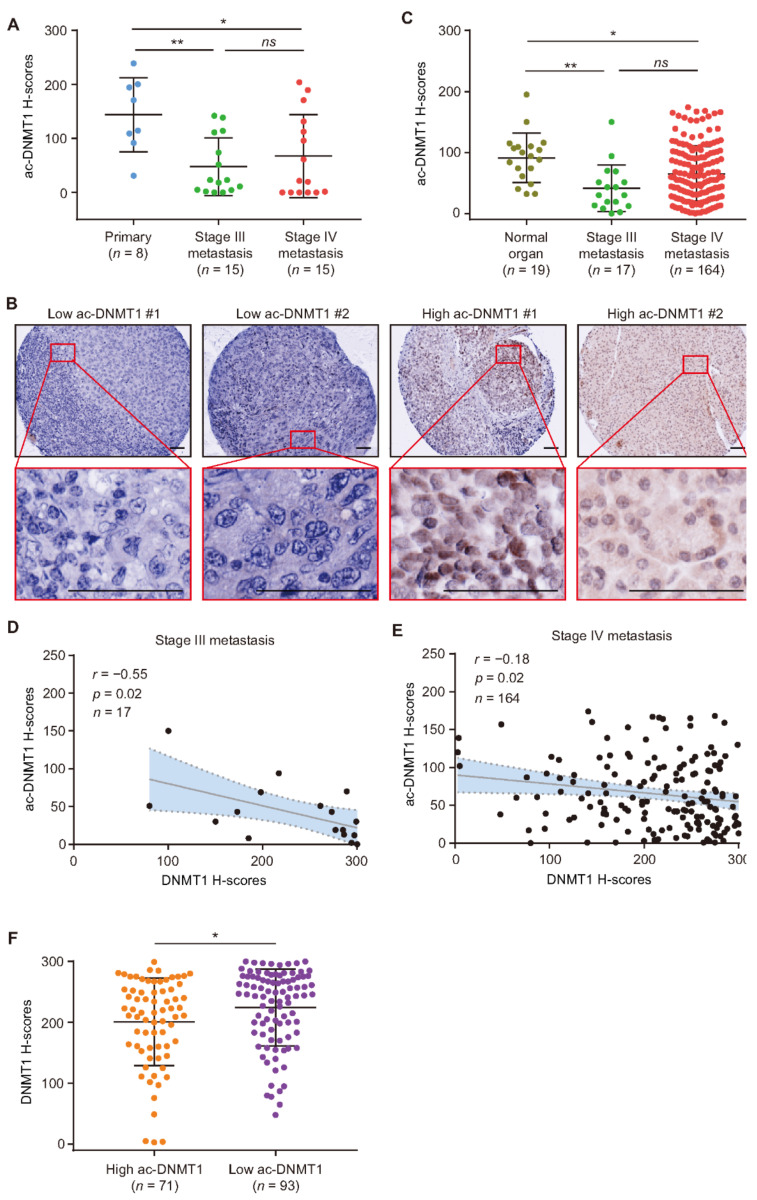
Decreased ac-DNMT1 protein levels are associated with melanoma progression. (**A**) Comparison of ac-DNMT1 H-scores in FFPE tissues from primary melanoma, stage III, and stage IV metastatic FFPE tissues. (**B**,**C**) Representative IHC images and H-score quantification of ac-DNMT1 in normal organ tissues, stage III, and stage IV metastasis from TMA cohort. Scale bars = 20 μm. (**D**,**E**) Correlation between ac-DNMT1 and DNMT1 IHC H-scores in stage III (**D**) and stage IV(**E**) metastatic FFPE tissues. The best-fit line (straight line) and the 95% CI (dotted line) are shown in grey. (**F**) Comparison of DNMT1 H-scores in stage IV patients with high or low ac-DNMT1 levels. Data represent the mean ± SD. *ns*: not significant, * *p* < 0.05, and ** *p* < 0.01.

To determine the protein levels of ac-DNMT1, IHC analysis was performed on FFPE tissues including primary melanoma, stage III, and stage IV metastatic melanoma. The results demonstrated a significant reduction in ac-DNMT1 protein levels in metastatic melanoma (stage III mean H-score = 48, *p* = 0.006; stage IV mean H-score = 67, *p* = 0.03) compared to primary melanoma tissues (mean H-score = 144, Figure 3A). We then verified the results by performing IHC for ac-DNMT1 in the melanoma TMA (Figure 3B). Ac-DNMT1 protein levels were significantly reduced in stage III (mean H-score = 41, *p* = 0.001) and stage IV (mean H-score = 65, *p* = 0.03) melanoma tissues compared to normal organ tissues (mean H-score = 91, Figure 3C). Most importantly, a significant inverse correlation was observed between ac-DNMT1 and DNMT1 protein levels in both stage III (*r* = −0.55, *p* = 0.02; Figure 3D) and stage IV (*r* = −0.18, *p* = 0.02; Figure 3E) melanoma patients. Consistently, stage IV patients with low ac-DNMT1 levels showed significantly higher DNMT1 protein levels than patients with high ac-DNMT1 (*p* = 0.02; Figure 3F). These results indicated that ac-DNMT1 is inversely correlated with DNMT1 protein levels and is associated with melanoma disease progression. Thus, ac-DNMT1 may have major implications in controlling DNMT1 protein expression.

### 3.3. Downregulation of Ac-DNMT1 Is Associated with TIP60 Reduction in Melanoma

**Figure 4:**
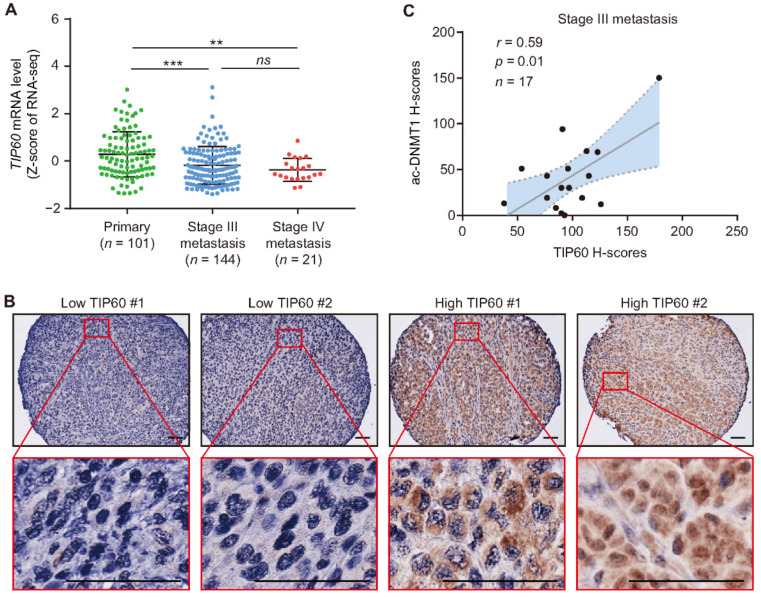

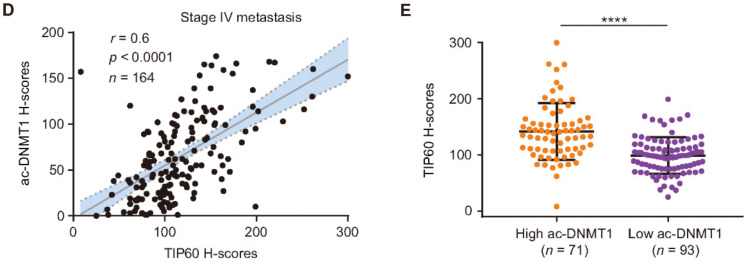
Ac-DNMT1 and TIP60 protein levels positive correlate in metastatic melanoma. (**A**) Comparison of *TIP60* mRNA expression using TCGA SKCM dataset. (**B**) Representative IHC images of TIP60 in the TMA melanoma cohort. Scale bars = 20 μm. (**C**,**D**) Correlation between TIP60 and ac-DNMT1 protein levels in stage III (**C**) and stage IV (**D**) metastatic FFPE tissues. (**E**) Comparison of TIP60 H-scores in stage IV patients with high and low ac-DNMT1 protein levels. The best-fit line (straight line) and the 95% CI (dotted line) are shown in grey. Data represent the mean ± SD. *ns*: not significant, ** *p* < 0.01, *** *p* < 0.001, and **** *p* < 0.0001.

Then, the mRNA expression levels of four genes (*TIP60*, *USP7*, *HDAC1*, and *UHRF1*) controlling the main PTMs occurring in DNMT1 were assessed in the TCGA SKCM dataset. A significant decrease in *TIP60* mRNA levels were observed in stage III (mean Z-score = −0.18, *p* = 0.0003) and stage IV metastasis (mean Z-score = −0.37, *p* = 0.007) compared to primary melanomas (mean Z-score = 0.28, Figure 4A). No significant differences were found for *USP7*, *HDAC1*, and *UHRF1* at the mRNA level using the TCGA SKCM database (Figure S2A–C). TIP60 acetylates DNMT1 and promotes its proteasomal-mediated degradation [28,34,45]. We performed IHC analysis for TIP60 on our SJCI TMA cohort (Figure 4B). There were significant positive correlations between TIP60 and ac-DNMT1 protein levels in tissues from both stage III (*r* = 0.59, *p* = 0.01) and stage IV (*r* = 0.6, *p* < 0.0001) melanoma patients (Figure 4C,D). Stage IV patients with low ac-DNMT1 protein levels showed significantly lower TIP60 protein levels compared to patients with high ac-DNMT1 (*p* < 0.0001; Figure 4E). Taken together, these results indicate that TIP60 and ac-DNMT1 protein expression positively correlate. *TIP60* mRNA downregulation during melanoma progression explains the reduction in TIP60 protein levels and the consequent reduction in ac-DNMT1 protein levels observed in metastatic melanoma.

### 3.4. Downregulation of Ac-DNMT1 Is Associated with a USP7 Reduction in Melanoma

**Figure 5:**
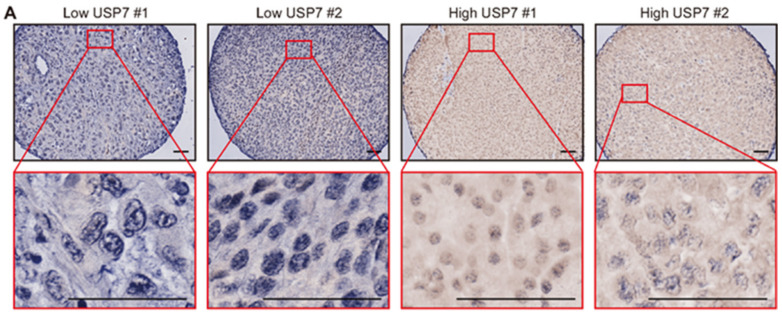

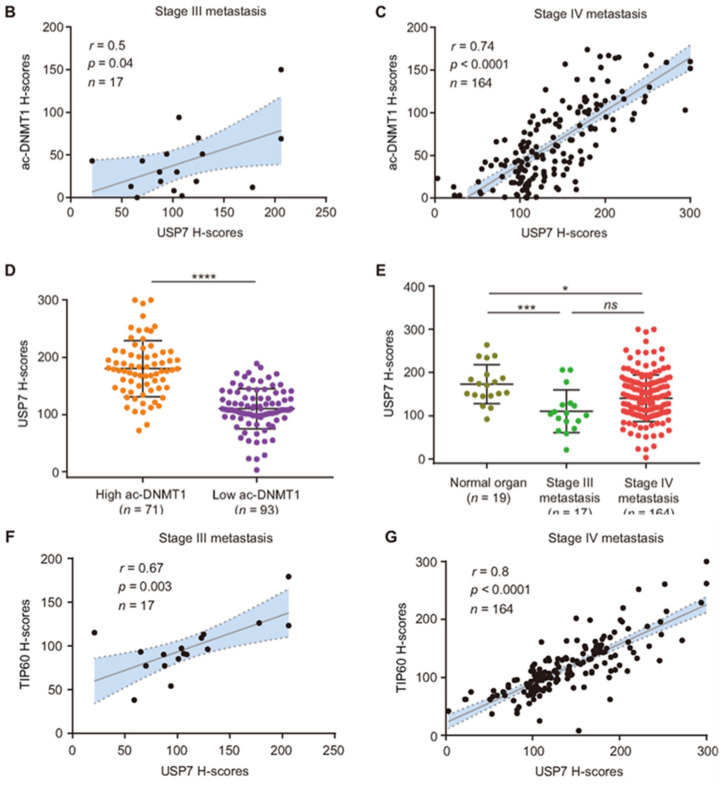
Ac-DNMT1 and USP7 protein levels positive correlate in metastatic melanoma. (**A**) Representative IHC images of USP7 in the TMA melanoma cohort. Scale bars = 20 μm. (**B**,**C**) Correlation between USP7 and ac-DNMT1 protein levels in stage III (**B**) or stage IV (**C**) metastatic FFPE tissues. (**D**) Comparison of USP7 H-scores in stage IV patients with high or low ac-DNMT1 protein levels. (**E**) Comparison of USP7 H-scores in normal organ tissues, stage III, and stage IV metastasis from the TMA melanoma cohort. (**F**,**G**) Correlation between USP7 and TIP60 protein levels in stage III (**F**) or stage IV (**G**) metastatic FFPE tissues. The best-fit line (straight line) and the 95% CI (dotted line) are shown in grey. Data represent the mean ± SD. *ns*: not significant, * *p* < 0.05, *** *p* < 0.001, and **** *p* < 0.0001.

In pancreatic cancer cells, USP7 regulation of DNMT1 stability is dependent on DNMT1 acetylation [34]. USP7 belongs to the peptidase C19 family and deubiquitinates proteins such as p53 and DNMT1 [46]. We therefore examined the USP7 protein levels to understand the relationship between DNMT1 and ac-DNMT1 as related to TIP60 and melanoma progression. IHC analysis was assessed for USP7 on the SJCI TMA cohort (Figure 5A). There were significant positive correlations between USP7 and ac-DNMT1 protein levels in both stage III (*r* = 0.5, *p* = 0.04) and stage IV (*r* = 0.74, *p* < 0.0001) metastatic melanoma samples (Figure 5B,C). Among stage IV melanoma, patients with low ac-DNMT1 protein levels showed significantly lower USP7 protein levels compared to patients with high ac-DNMT1 (*p* < 0.0001; Figure 5D). Consistent with a reduction in ac-DNMT1 protein level during melanoma disease progression (Figure 3A,C), a significant decrease in USP7 protein levels were observed in stage III (mean H-score = 110, *p* = 0.0006) and stage IV metastasis (mean H-score = 140, *p* = 0.02) compared to normal organ tissues (mean H-score = 173, Figure 5E). Additionally, we observed a significant positive correlation between TIP60 and USP7 (*r* = 0.67, *p* = 0.003 for stage III; *r* = 0.8, *p* < 0.0001 for stage IV) protein levels in metastatic melanoma (Figure 5F,G). These findings demonstrate the relation of TIP60 and USP7 in controlling DNMT1 acetylation and protein stability in cutaneous melanoma tissues, respectively.

### 3.5. Ac-DNMT1 Protein Levels Negatively Correlated with Ki-67 in Metastatic Melanoma

**Figure 6:**
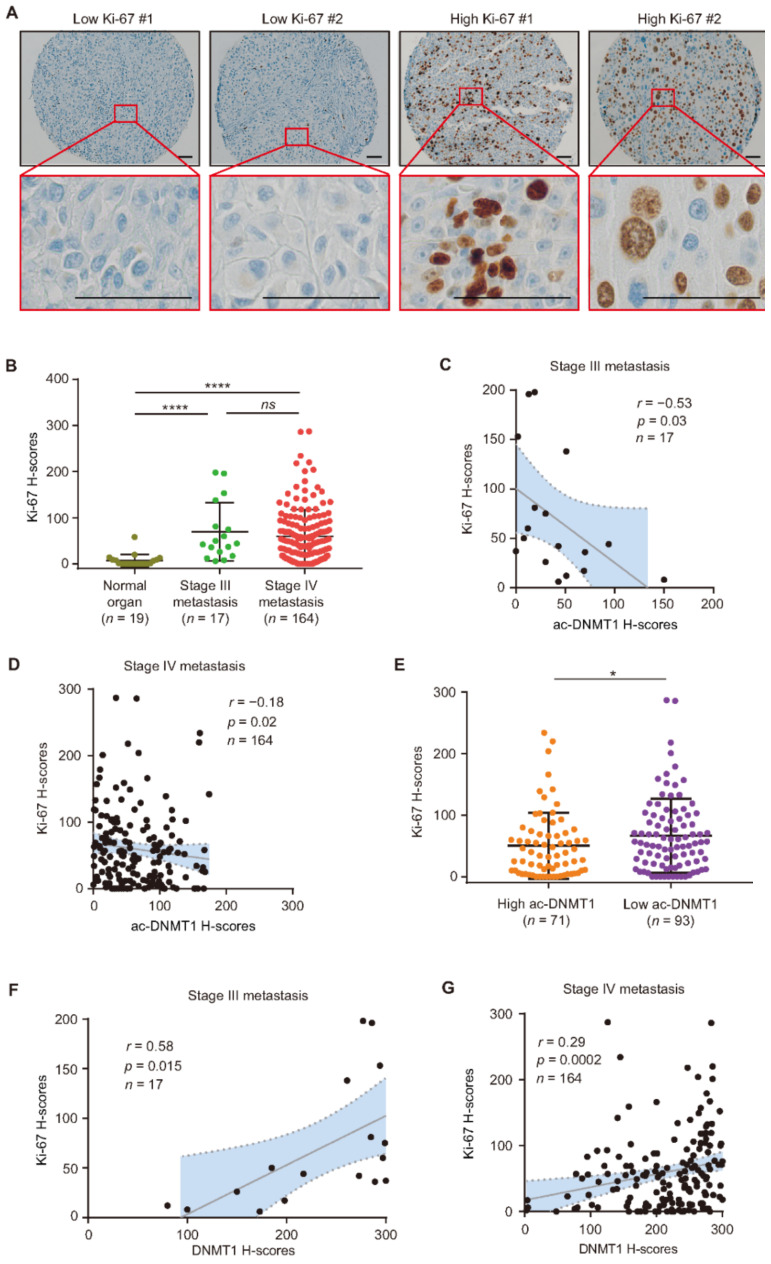
Ac-DNMT1 and Ki-67 protein levels correlate in metastatic melanoma. (**A**,**B**) Representative IHC images and H-score quantification of Ki-67 in normal organ tissues, stage III, and stage IV metastatic melanomas from the TMA cohort. Scale bars = 20 μm. (**C**,**D**) Correlation between Ki-67 and ac-DNMT1 H-scores in stage III (**C**) or stage IV (**D**) metastatic FFPE tissues. (**E**) Comparison of Ki-67 H-scores in stage IV patients with high or low ac-DNMT1 levels. (**F**,**G**) Correlation between Ki-67 and DNMT1 IHC H-scores in stage III (**F**) or stage IV (**G**) metastatic FFPE tissues. The best-fit line (straight line) and the 95% CI (dotted line) are shown in grey. Data represent the mean ± SD. *ns*: not significant, * *p* < 0.05, and **** *p* < 0.0001.

To validate the role of ac-DNMT1 in relation to melanoma disease progression and tumor growth, the protein expression of the proliferation protein marker Ki-67 was assessed on the SJCI TMA cohort (Figure 6A). As expected, Ki-67 protein expression was significantly increased in stage III (mean H-score = 69, *p* < 0.0001) and stage IV (mean H-score = 60, *p* < 0.0001) metastatic melanoma tissues compared to normal organ tissues (mean H-score = 7, Figure 6B). Most importantly, there were significant inverse correlations between ac-DNMT1 and Ki-67 protein levels in both stage III (*r* = −0.53, *p* = 0.03, Figure 6C) and stage IV metastatic melanoma tissues (*r* = −0.18, *p* = 0.02, Figure 6D). Stage IV patients with low ac-DNMT1 protein levels showed significantly higher Ki-67 protein levels compared to patients with high ac-DNMT1 (*p* = 0.039; Figure 6E). These findings were consistent with the significant positive correlations between DNMT1 and Ki-67 protein levels in both stage III (*r* = 0.58, *p* = 0.015, Figure 6F) and stage IV (*r* = 0.29, *p* = 0.0002, Figure 6G) melanoma patients. The results indicate that metastatic melanoma tumors with low ac-DNMT1 and high DNMT1 levels showed higher doubling rate.

### 3.6. Reduced Ac-DNMT1 Protein Levels Are Associated with Poor Melanoma-Specific Survival

**Figure 7:**
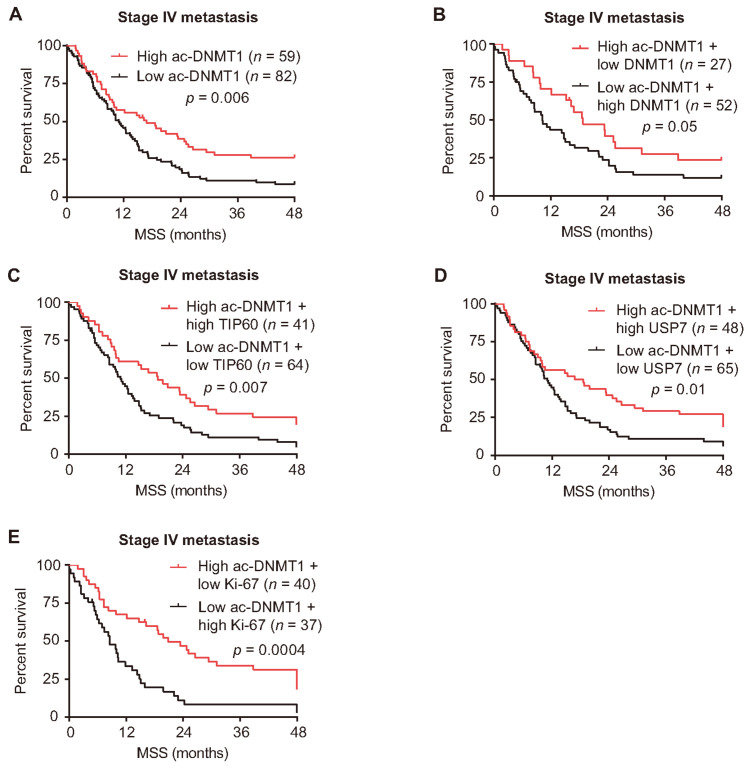
Low ac-DNMT1 protein level is a predictive factor for poor MSS. (**A**) MSS curves for stage IV metastasis from the TMA cohort. Patients were divided according to the mean IHC H-scores of ac-DNMT1. (**B**) MSS curves for stage IV metastasis from the TMA cohort. Patients were divided according to the mean IHC H-scores of ac-DNMT1 together with DNMT1 (**B**), TIP60 (**C**), USP7 (**D**), and Ki-67 (**E**) into high and low groups, respectively.

To examine the clinical associations between survival outcomes and ac-DNMT1 protein levels, stage IV melanoma patients were stratified based on the mean H-scores into patients with low versus high ac-DNMT1 protein expression. A total of 141 patients were analyzed for ac-DNMT1 (Table S1). The same methodology was utilized to determine associations between DNMT1, TIP60, and USP7 protein expression with survival outcomes (Table S1). A significant correlation was found between ac-DNMT1 protein levels with disease outcomes status in stage IV melanoma patients. Up to 63% (74 of 117) of the melanoma patients who expired had low ac-DNMT1 protein levels, while 33% (8 of 24) of the alive patients had low ac-DNMT1 protein levels (*p* = 0.007, χ^2^ test; Table S2). The Kaplan–Meier analysis showed that low ac-DNMT1 protein levels are associated with shorter 4-year MSS (*p* = 0.006, log-rank test; Figure 7A) in stage IV melanoma patients. Patients with concurrent high ac-DNMT1 and low DNMT1 protein levels had a better prognosis compared to patients with low ac-DNMT1 and high DNMT1 (log-rank test *p* = 0.05; Figure 7B). Consistently, stage IV patients with high ac-DNMT1 combined with high TIP60, high USP7, or low Ki-67 protein levels had a better prognosis compared to patients with low ac-DNMT1 combined with low TIP60, low USP7, or high Ki-67 protein levels (log-rank test *p* = 0.007, *p* = 0.01, and *p* = 0.0004 respectively; Figure 7C–E). Our results indicated that low ac-DNMT1 protein levels are significantly associated with a poor MSS in patients with stage IV melanoma.

### 3.7. Ac-DNMT1 Protein Level Is an Independent Prognostic Factor for Melanoma Metastasis

To evaluate the prognostic significance of our findings, we performed multivariate Cox regression analyses with known prognostic factors to investigate whether ac-DNMT1 protein level is an independent prognostic biomarker for 4-year MSS in stage IV patients. We assessed 141 stage IV metastatic melanoma patients with age, gender, organ site, and ac-DNMT1/DNMT1/TIP60/USP7 H-scores in a regression model (Table 1, Table 2, Table 3 and Table 4). The results revealed that ac-DNMT1 protein level is a significant and independent factor associated with MSS (HR, 0.994; 95% confidential interval (CI), 0.990–0.998; *p* = 0.002; Table 1). Additionally, ac-DNMT1 protein level is a significant independent prognostic factor when Ki-67 protein level was included in the multivariate analysis (HR, 0.995; 95% CI, 0.991–0.999; *p* = 0.009; Table 5). These findings indicate that ac-DNMT1 protein levels may represent a promising biomarker to predict MSS in stage IV melanoma patients.

## 4. Discussion

DNMT1 is an important epigenetic regulator that plays a key role in the maintenance of DNA methylation during the S phase of the cell cycle [47]. The aberrant DNA methylation of genes is an epigenetic hallmark of many cancers including cutaneous melanoma [11,17,47,48,49]. Our study identified the association between DNMT1 and ac-DNMT1 protein levels in tumor progression using a clinically annotated TMA. DNMT1 stability is mediated by acetylation and ubiquitination through coordinated actions of regulatory proteins. As previously demonstrated, TIP60 and HDAC1 balance the acetylation levels of DNMT1 [26]. Meanwhile, ubiquitin-like with PHD and ring finger domains 1 (UHRF1) and USP7 have been reported to play a role in DNMT1 ubiquitination or deubiquitination processes, respectively [26,28,35]. The role of ubiquitination in DNMT1 degradation is an important regulatory event as it reflects the changes in DNMT1 level during the cell cycle status [50].

Previous studies demonstrate that acetylation of DNMT1 by TIP60 impairs DNMT1–USP7 interaction and stimulates the proteasomal degradation of DNMT1 [34]. Additionally, acetylation of Lys1349 and Lys1415 in the catalytic domain of DNMT1 affects the methyltransferase activity of DNMT1 [51]. The histone deacetylase HDAC1 plays an important role in DNMT1 stability by deacetylating the lysine residues [52,53]. Panobinostat is an approved anti-cancer chemotherapy drug classified as a non-selective HDAC inhibitor [52,54]. After panobinostat treatment, we observed an accumulation of ac-DNMT1 and reduced DNMT1 protein expression in melanoma cell lines. These results further supported the inverse correlation observed between DNMT1 and ac-DNMT1 protein levels using the TMA IHC analysis of metastatic melanoma patients.

Additionally, we identified the positive correlations between TIP60/USP7 and ac-DNMT1 protein levels in stage IV melanoma metastasis. This is consistent with their functional roles in controlling DNMT1 stability, and further supporting the inverse correlation between ac-DNMT1 and DNMT1 protein levels observed in the TMA IHC analysis. The decreased expression levels of TIP60 or USP7 were associated with melanoma progression, which suggested these two proteins play an important role in modulating the levels of ac-DNMT1 in melanoma metastasis. Our observations from melanoma patient tumors are consistent with previous reports on cell lines from other tumor types. Du et al. found that the overexpression of TIP60 reduced the abundance of endogenous DNMT1 in embryonic kidney immortalized HEK293 cell lines, while knockdown of TIP60 increased DNMT1 in the colorectal carcinoma HCT116 cell lines [26]. Similarly, Ashraf et al. reported that reduced DNA methylation and downregulated DNMT1 were observed in TIP60-eGFP transfected HeLa cell lines [55].

There are two studies in different cancer types showing diverging results for DNMT1. In breast cancer [56], high DNMT1 expression is significantly associated with poor survival (*p* < 0.05) using multivariate analysis. However, head and neck squamous cell carcinoma (HNSCC) with advanced-grade tumors have high DNMT1 expression that is associated with better overall survival [57]. In our study, 4-year MSS in stage IV patients revealed that high ac-DNMT1 protein level, not DNMT1 protein expression, is a significant independent prognostic marker in a multivariate analysis. Our study showed a significant downregulation of ac-DNMT1 protein levels that were inversely correlated with Ki-67 protein levels. Whether the ac-DNMT1 protein levels and disease outcomes are context dependent on the cancer type requires further investigation. Future pan-cancer studies may lead to better understanding and could help determine whether these findings represent conserved molecular mechanisms controlling DNMT1 protein expression.

Taken together, our studies demonstrated ac-DNMT1 is a promising melanoma prognostic factor for predicting MSS. The findings of the relation between ac-DNMT1 and other biomarkers may help to optimize treatment strategies of stage IV metastasis. Further studies in a large multicenter study will be needed to validate the findings.

## 5. Conclusions

In this study, we systematically investigated the translational utility of DNMT1/ac-DNMT1 protein levels in cutaneous metastatic melanoma. Using a TMA, we observed a significant decrease in ac-DNMT1 protein levels, and an inverse correlation between ac-DNMT1 and DNMT1 protein levels in metastatic melanoma patients. These events were shown to be due to the downregulation of TIP60/USP7 protein expression in advanced stage melanoma metastasis. Kaplan–Meier and multivariate Cox proportional hazard analysis revealed that ac-DNMT1 protein level is a significant independent prognostic factor for 4-year MSS in stage IV melanoma patients.

## Figures and Tables

**Table 1:** Multivariate analyses including ac-DNMT1 protein levels for 4-year MSS ^1^ in stage IV melanoma patients.

Variable	Multivariate Analysis		
	*p*-Value	HR ^2^	95% CI ^3^
Ac-DNMT1 H-score	0.002	0.994	0.990–0.998
Age	0.009	0.983	0.971–0.996
Gender	0.705	1.082	0.719–1.629
Organ site	0.025	0.841	0.722–0.978

^1^ MSS, melanoma-specific survival; ^2^ HR, hazard ratio; ^3^ CI, confidence interval.

**Table 2:** Multivariate analyses including DNMT1 protein level for 4-year MSS ^1^ in stage IV melanoma patients.

Variable	Multivariate Analysis		
	*p*-Value	HR ^2^	95% CI ^3^
DNMT1 H-score	0.566	1.001	0.998–1.004
Age	0.012	0.984	0.971–0.996
Gender	0.324	1.229	0.816–1.850
Organ site	0.015	0.829	0.712–0.965

^1^ MSS, melanoma-specific survival; ^2^ HR, hazard ratio; ^3^ CI, confidence interval.

**Table 3:** Multivariate analyses including TIP60 protein levels for 4-year MSS ^1^ in stage IV melanoma patients.

Variable	Multivariate Analysis		
	*p*-Value	HR ^2^	95% CI ^3^
TIP60 H-score	0.170	0.763	0.519–1.123
Age	0.010	0.983	0.971–0.996
Gender	0.598	1.118	0.738–1.695
Organ site	0.027	0.841	0.721–0.980

^1^ MSS, melanoma-specific survival; ^2^ HR, hazard ratio; ^3^ CI, confidence interval.

**Table 4:** Multivariate analyses including USP7 protein levels for 4-year MSS ^1^ in stage IV melanoma patients.

Variable	Multivariate Analysis		
	*p*-Value	HR ^2^	95% CI ^3^
USP7 H-score	0.111	0.997	0.994–1.001
Age	0.016	0.985	0.972–0.997
Gender	0.555	1.131	0.751–1.705
Organ site	0.031	0.844	0.723–0.984

^1^ MSS, melanoma-specific survival; ^2^ HR, hazard ratio; ^3^ CI, confidence interval.

**Table 5:** Multivariate analyses including ac-DNMT1 and Ki-67 protein levels for 4-year MSS ^1^ in stage IV melanoma patients.

Variable	Multivariate Analysis		
	*p*-Value	HR ^2^	95% CI ^3^
Ac-DNMT1 H-score	0.009	0.995	0.991–0.999
Ki-67 H-score	0.026	1.549	1.055–2.276
Age	0.008	0.982	0.970–0.995
Gender	0.429	1.186	0.777–1.811
Organ site	0.020	0.835	0.717–0.972

^1^ MSS, melanoma-specific survival; ^2^ HR, hazard ratio; ^3^ CI, confidence interval.

## Data Availability

Data is available in a publicly accessible repository. TCGA SKCM datasets (Firehose Legacy source) for copy number variation, mRNA expression, and the corresponding clinical information were obtained in February 2019 from https://www.cbioportal.org/ (accessed on 20 June 2021). The Talantov’s melanoma microarray data [58] and Xu’s melanoma microarray data [59] presented in this study are openly available in the GEO database at GSE3189 and GSE8401, respectively. IHC data is contained within the article or Supplementary Materials.

## Supplementary Materials

The following are available online at https://www.mdpi.com/article/10.3390/cancers13184691/s1, Figure S1: Uncropped Western blot images for Figure 2C,D, Figure S2: Comparison of USP7 (A) HDAC1 (B) UHRF1 (C) mRNA expression in primary, stage III, and stage IV metastatic melanoma from TCGA SKCM dataset, Table S1: Clinicopathological characteristics and H-score values detected by IHC for stage IV melanoma patients, Table S2: Associations between clinicopathological characteristics and H-score values detected by IHC for stage IV melanoma patients.
