# Supplementary material for: Acetylated DNMT1 Downregulation and Related Regulatory Factors Influence Metastatic Melanoma Patients Survival
Source: Cancers (Basel). 2021 Sep 18;13(18):4691. doi: 10.3390/cancers13184691 (PMC8471314; doi:10.3390/cancers13184691)
Supplement: Supplementary file 1 [file cancers-13-04691-s001.zip › cancers-1369578-supplementary.pdf]

cancers

MDPI

Supplementary Materials: Acetylated DNMT1 Downregulation and Related Regulatory Factors Influence Metastatic Melanoma Patients Survival

Xiaoqing Zhang, Matias A. Bustos, Yoshiaki Shoji, Romela Irene Ramos, Yuuki Iida, Rebecca Gentry, Teh-Ling Takeshima and Dave S. B. Hoon

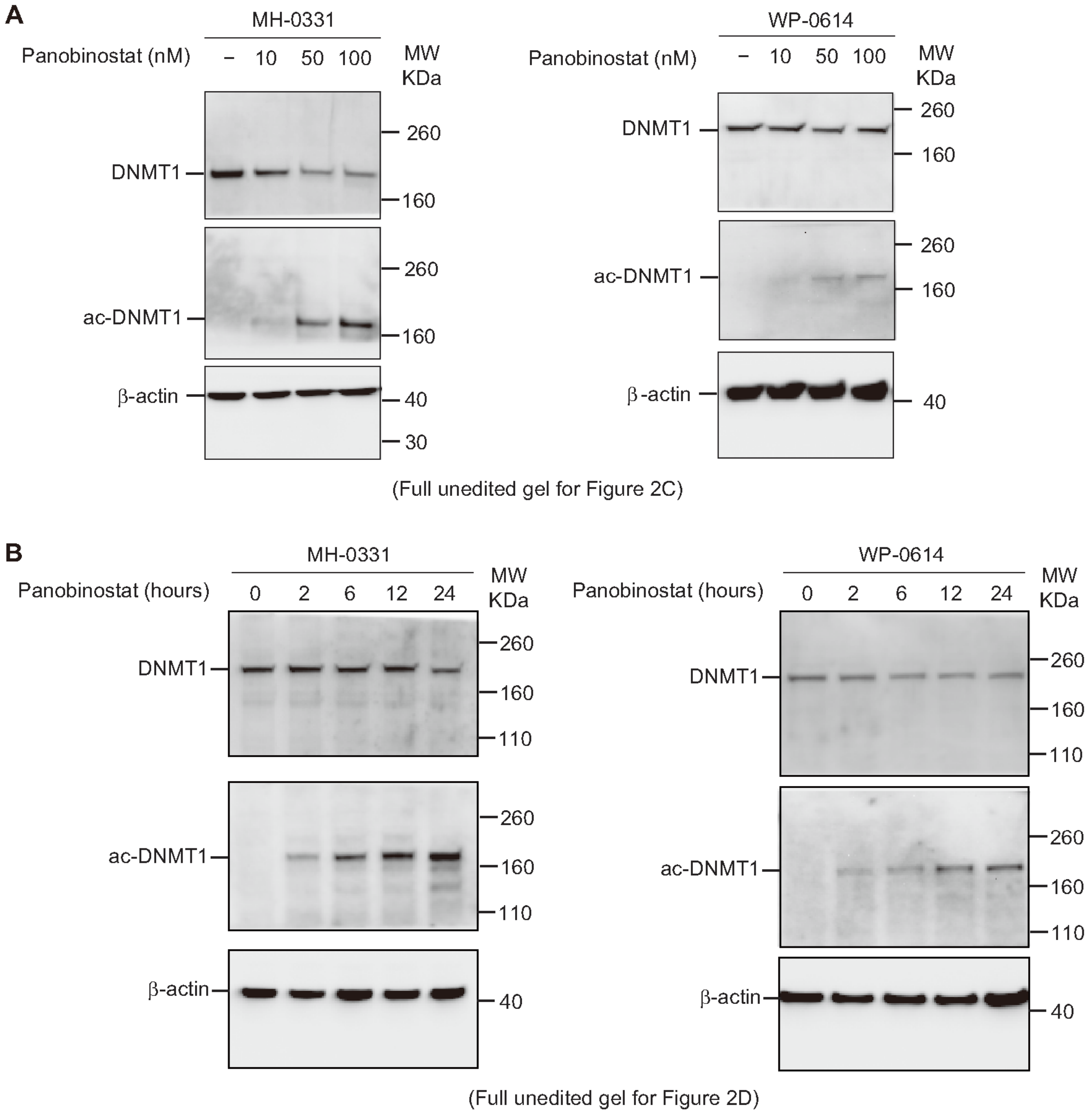

Figure S1. Uncropped western blot images for **Figures 2C** and **D**.

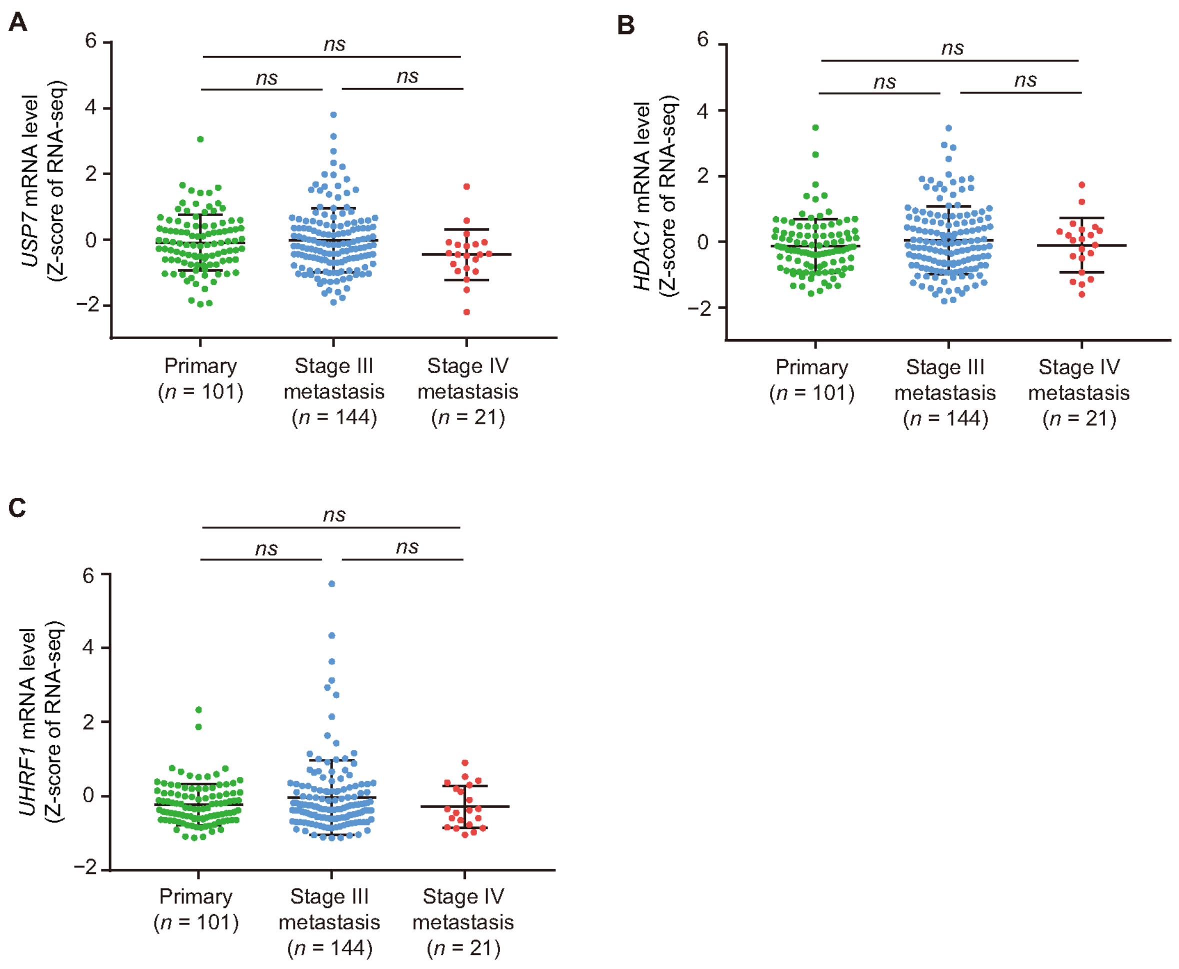

Figure S2. Comparison of *USP7* (**A**) *HDAC1* (**B**) *UHRF1* (**C**) mRNA expression in primary, stage III, and stage IV metastatic melanoma from TCGA SKCM dataset. Data represent the mean ± SD. *ns*: not significant.

Table S1. Clinicopathological characteristics and H-score values detected by IHC for stage IV melanoma patients.

Variable	Factor	Number of cases (%)
Age (years)	<60	76 (54)
	≥60	65 (46)
Gender	Male	99 (70)
	Female	42 (30)
Organ site	Bowel	36 (26)
	Lung	26 (18)
	Distant skin	20 (14)
	Others	59 (42)
M stage [1]	M1a	26 (18)
	M1b	18 (13)
	M1c	67 (48)
	M1d	25 (18)
	NA	5 (3)
Survival status	Alive	24 (17)
	Expired	117 (83)
Ac-DNMT1 H-score (cutoff = mean)	Low	82 (58)
	High	59 (42)
DNMT1 H-score (cutoff = mean)	Low	57 (40)
	High	84 (60)
TIP60 H-score (cutoff = mean)	Low	82 (58)
	High	59 (42)
USP7 H-score (cutoff = mean)	Low	76 (54)
	High	65 (46)

[1] AJCC 8th Edition.

Table S2. Associations between clinicopathological characteristics and H-score values detected by IHC for stage IV melanoma patients.

Variable	Factor	ac-DNMT1			DNMT1			TIP60			USP7		
		Low cases	High cases	*p*-value	Low cases	High cases	*p*-value	Low cases	High cases	*p*-value	Low cases	High cases	*p*-value
**Age (years)**	<60	48	28	0.193	29	47	0.553	46	30	0.537	44	32	0.304
	≥60	34	31		28	37		36	29		32	33	
Gender	Male	56	43	0.557	38	61	0.448	50	49	0.005	48	51	0.048
	Female	26	16		19	23		32	10		28	14	
Organ site	Bowel	23	13	0.348	12	24	0.025	24	12	0.601	21	15	0.235
	Lung	12	14		17	9		15	11		17	9	
	Distant skin	14	6		9	11		12	8		12	8	
	Others	33	26		19	40		31	28		26	33	
M stage	M1a	18	8	0.349	12	14	0.212	18	8	0.425	15	11	0.771
	M1b	8	10		11	7		8	10		10	8	
	M1c	37	30		25	42		38	29		33	34	
	M1d	16	9		8	17		15	10		15	10	
	NA	5											
Survival status	Alive	8	16	0.007	8	16	0.437	12	12	0.374	9	15	0.077
	Expired	74	43		49	68		70	47		67	50	
